# *Research 101:* An online course introducing medical students to research

**DOI:** 10.1017/cts.2022.435

**Published:** 2022-08-01

**Authors:** Jason T. Blackard, Jacqueline M. Knapke, Patrick H. Ryan, Stephanie Schuckman, Jennifer Veevers, William D. Hardie, James E. Heubi

**Affiliations:** 1 Division of Digestive Diseases, Department of Internal Medicine, University of Cincinnati College of Medicine, Cincinnati, OH, USA; 2 Center for Clinical and Translational Science and Training, University of Cincinnati, Cincinnati, OH, USA; 3 Division of Biostatistics and Epidemiology, Cincinnati Children’s Hospital, Cincinnati, OH, USA; 4 Department of Pediatrics, University of Cincinnati College of Medicine, Cincinnati, OH, USA

**Keywords:** Research training, medical student, scholar activity

## Abstract

**Introduction:**

Research is an important aspect of many medical students’ training. However, many medical students are not required to complete a scholarly project, and formal research training is often fragmented across the medical school curriculum. Thus, we developed an online, structured, asynchronous set of modules to introduce trainees to multiple topics relevant to the conduct of research.

**Methods:**

*Research 101* was piloted by 27 first-year medical students at the University of Cincinnati College of Medicine. Students’ knowledge, confidence, and satisfaction were assessed using a final quiz and pre- and post-module surveys with five-point Likert-scaled questions and open-ended text responses.

**Results:**

Pre-module survey results showed that learners felt most confident in *Conducting a literature search* and least confident in *Submitting an Institutional Review Board (IRB) protocol at UC*. Post-module mean scores were significantly increased compared to pre-module results for all modules and questions (*P* < 0.05). The response to “The content of this module met my needs” was high across all modules with 236 (84.0%) “yes” responses. Thematic analysis of open-ended text responses from post-module surveys identified several improvements to individual modules and to the overall structure of *Research 101*. A final quiz of 25 multiple choice questions covering content from all required modules was required. The median score was 21.

**Conclusions:**

Comparison of post-module to pre-module survey scores provided clear evidence of improved learning across all topics. The modules developed were responsive to the students’ needs, and students provided additional improvements for subsequent iterations of *Research 101*.

## Introduction

Research and scholarly pursuits are an important aspect of any medical student’s training. A meta-analysis of medical student research found that 72% of medical students were interested in performing research, and 31% of medical students were interested in a career that involved research [1]. Students who participated in research projects during medical school were 3.55 times more likely to report an interest in research as part of their future careers [1]. The 2018 Association of American Medical Colleges (AAMC) Medical School Graduation Questionnaire reported that 78.8% of medical school graduates participated in a research project with a faculty member compared to 69.3% in 2014 [2]. Overall, 50.5% of medical school graduates authored a peer-reviewed publication, and 44.4% wanted to be significantly involved in research in the future.

Medical students may conduct scholarly activity at any time during their training, including summer research electives, mandatory curricular activities, non-required/extracurricular research activities, and/or longitudinal research experiences. AAMC core competencies for entering medical students include thinking and reasoning competencies such as critical thinking, quantitative reasoning, scientific inquiry, and written communication [3]. Agmad *et al*. found that career advancement is a major motivation for performing research during medical school [1]. While there is a common student perception that research in medical school is necessary for a successful application to residency, Green *et al.* reported that program directors ranked research experience low among all selection criteria when all specialties were grouped together [4]. However, research experience was ranked highly among competitive specialties such as radiation oncology, plastic surgery, neurosurgery, and dermatology. Others suggest that rigorous training in the scientific method may increase student confidence in their clinical decision-making skills and enhance patient care [5]. Unfortunately, formal research training – when present – is often fragmented across the medical school curriculum. For example, Stone *et al*. reported that many medical schools did not incorporate research training or have an adequate focus on research, while others had no research curriculum at all or it was buried – not obvious – within the existing curriculum [6].

To reinforce basic research skills and fill gaps within the existing curriculum, we developed an online, structured, asynchronous set of modules – called *Research 101* – to introduce medical students to multiple topics that are relevant to the conduct of research. The objective of this report is to describe the *Research 101* curriculum and evaluate its effectiveness in a pilot study.

## Methods

To create *Research 101*, the primary author developed an initial list of topics based on experiences with research trainees at a variety of levels. These topics were then discussed with administrators, faculty, and staff working within the Center for Clinical and Translational Science and Training (CCTST), the Office of Student Affairs, the Office of Research, the Office of Medical Education, and the Office of Graduate Education at the University of Cincinnati College of Medicine (UCCOM). The overall course format and individual topics were also discussed with student members of the Research and Industry Relations Committee during the development process to ensure a student-centered approach. Final topics included: 1) getting started with *Research 101*, 2) introduction to research, 3) aligning expectations, 4) identifying a research mentor and a research project, 5) introduction to human subjects research and protections, 6) submitting an Institutional Review Board (IRB) protocol at UC, 7) conducting a literature search, 8) effective writing for publication, 9) transparency, rigor, and reproducibility in research, 10) study design and data analysis basics, 11) presenting your summer research, and 12) evaluating the literature and presenting a journal club. Additional topics were considered but were excluded because they were not appropriate for a general student audience, or they were placed in a “miscellaneous” module that could be added or revised in subsequent versions of *Research 101*.

**Fig. 1.**
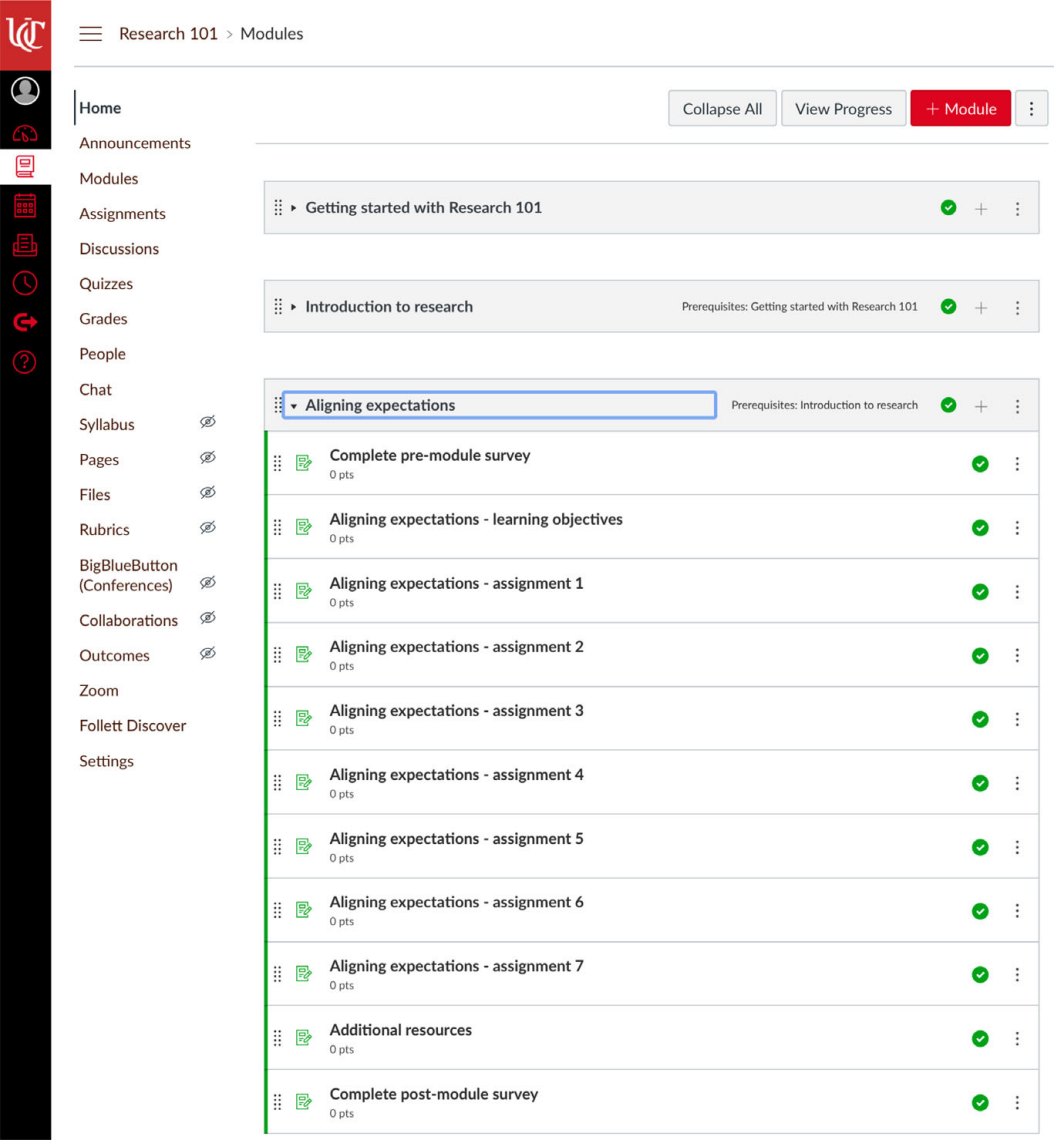
Content for the *Aligning expectations* module.

*Research 101* was designed as a zero-credit course through the University of Cincinnati. This option allows students to register and to receive documentation of their participation in the form of a transcript but does not require changing a degree program’s curriculum or charging students tuition. The modules were offered asynchronously through the online educational platform Canvas (Salt Lake City, UT), so that learners could participate at their own pace. The first module – Getting started with *Research 101* – provided a brief introduction to the course and its content, including how to log into Canvas, where direct technical questions about the educational platform, and how to communicate with the instructor about content-specific questions. Each module consisted of several elements including learning objectives, assignments, a pre-module survey, and a post-module survey. Additional resources were also included for some modules. The assignments typically involved one or more activities including watching a prerecorded lecture, watching a YouTube video, reading the content of a website, reading a publication or report, or responding to discussion questions. Specific attention was given to utilizing existing resources such as websites, previously developed case studies, and/or existing videos so that the content would not be specific to one learner type or a single institution. Hyperlinks were utilized extensively throughout the modules to provide direct access to publicly available resources. Figure 1 shows all content for the *Aligning expectations* module to illustrate the overall structure of an individual module within Canvas. By clicking on the arrow to the left of the module title, participants can view all content within a particular module. For example, the *Aligning expectations* module included pre- and post-module surveys, learning objectives, seven assignments labeled with the module title and the assignment number, as well as additional resources. Learning objectives for all modules are provided in Table 1.

**Table 1.** Learning objectives for the *Research 101* modules

**Getting started with** ***Research 101*** Learn how to log into the *Research 101* series through Canvas Understand the structure of a *Research 101* module Learn where to go with questions about a *Research 101* module
**Introduction to research** Define research Identify various types of research Recognize the stages of clinical/translational research Identify the steps of the scientific method
**Aligning expectations** Identify your skills as a mentee/trainee Identify possible expectations of a mentee Identify possible expectations of a mentor Describe possible barriers to an effective mentor–mentee relationship
**Identifying a research mentor and a research project** Describe the characteristics of your ideal mentor List resources to identify an appropriate mentor and to develop a research project Strategize possible solutions to common barriers to an effective mentor–mentee relationship Understand how to write a clear and concise research project description
**Introduction to human subjects research and protections** Understand the history of human subjects research Define human subjects research List the requirements for protecting human subjects in research
**Submitting an Institutional Review Board (IRB) protocol at UC** List resources for submitting an IRB protocol for review List the components of an IRB submission Understand common issues with IRB submissions and strategize possible solutions List resources for the protection of human subjects, biosafety, and animal care and training on these topics
**Conducting a literature search** Describe the use of PubMed for literature searches Describe the use of Google Scholar for literature searches List resources for conducting an effective literature search
**Effective writing for publication** List the various types of publications Describe the elements of a manuscript Describe an effective abstract List resources for improving ones writing skills
**Transparency, rigor, and reproducibility in research** Describe the need for transparency and its impact on the research process Describe the need for rigor and reproducibility and their impact on the research process Identify approaches to enhance the transparency, rigor, and reproducibility of your research project
**Study design and data analysis basics** List different types of epidemiologic studies Define key characteristics and limitations of cross-sectional studies Define key characteristics and limitations of cohort studies List resources for study design and data analysis
**Presenting your summer research** Describe elements of an effective poster List venues for presenting research projects Describe elements of an effective lab meeting presentation
**Evaluating the literature and presenting a journal club** Describe strategies for reading and critiquing a scientific article Describe elements of an effective journal club
**Final quiz**

**Fig. 2.**
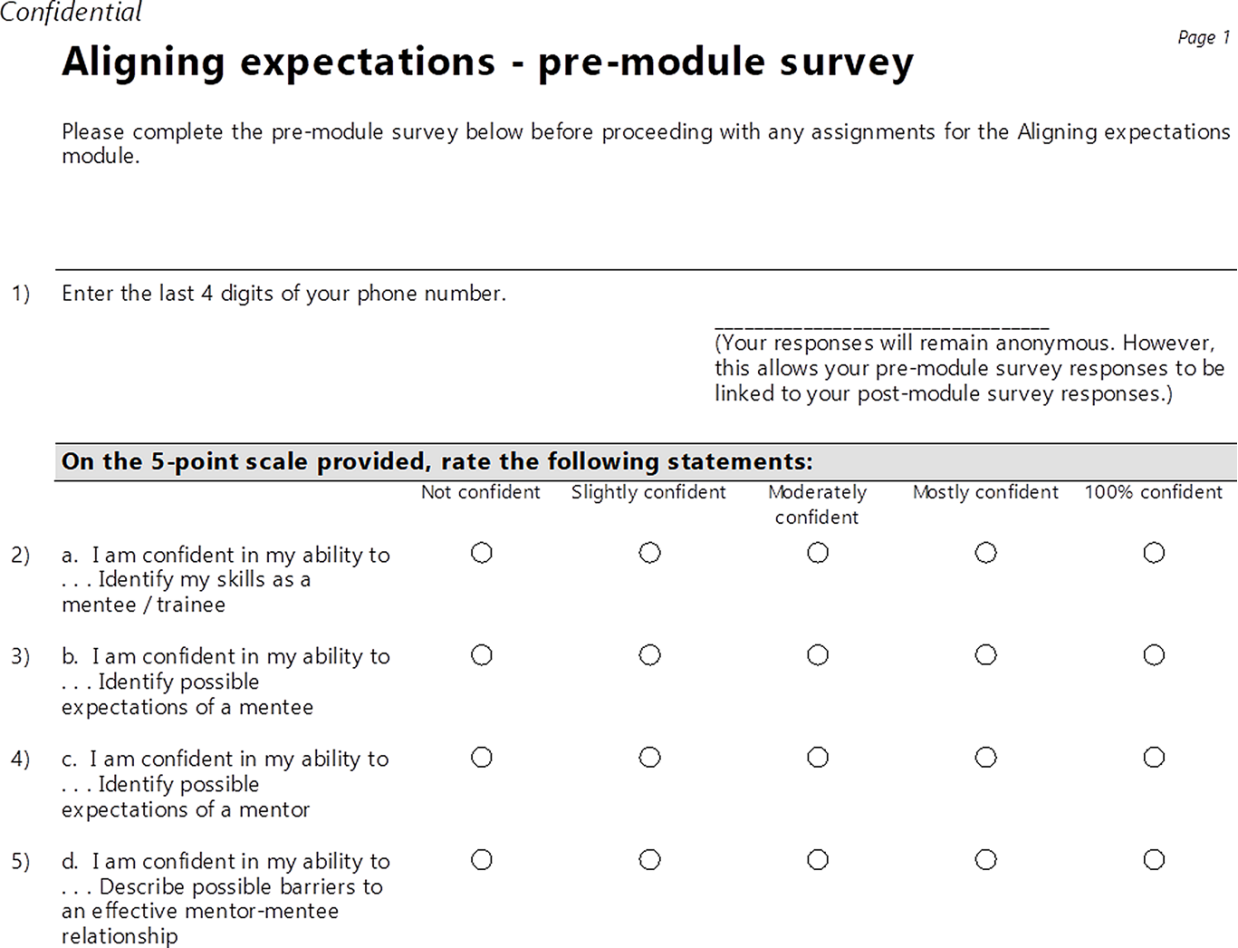
Pre-module survey for the *Aligning expectations* module.

**Fig. 3.**
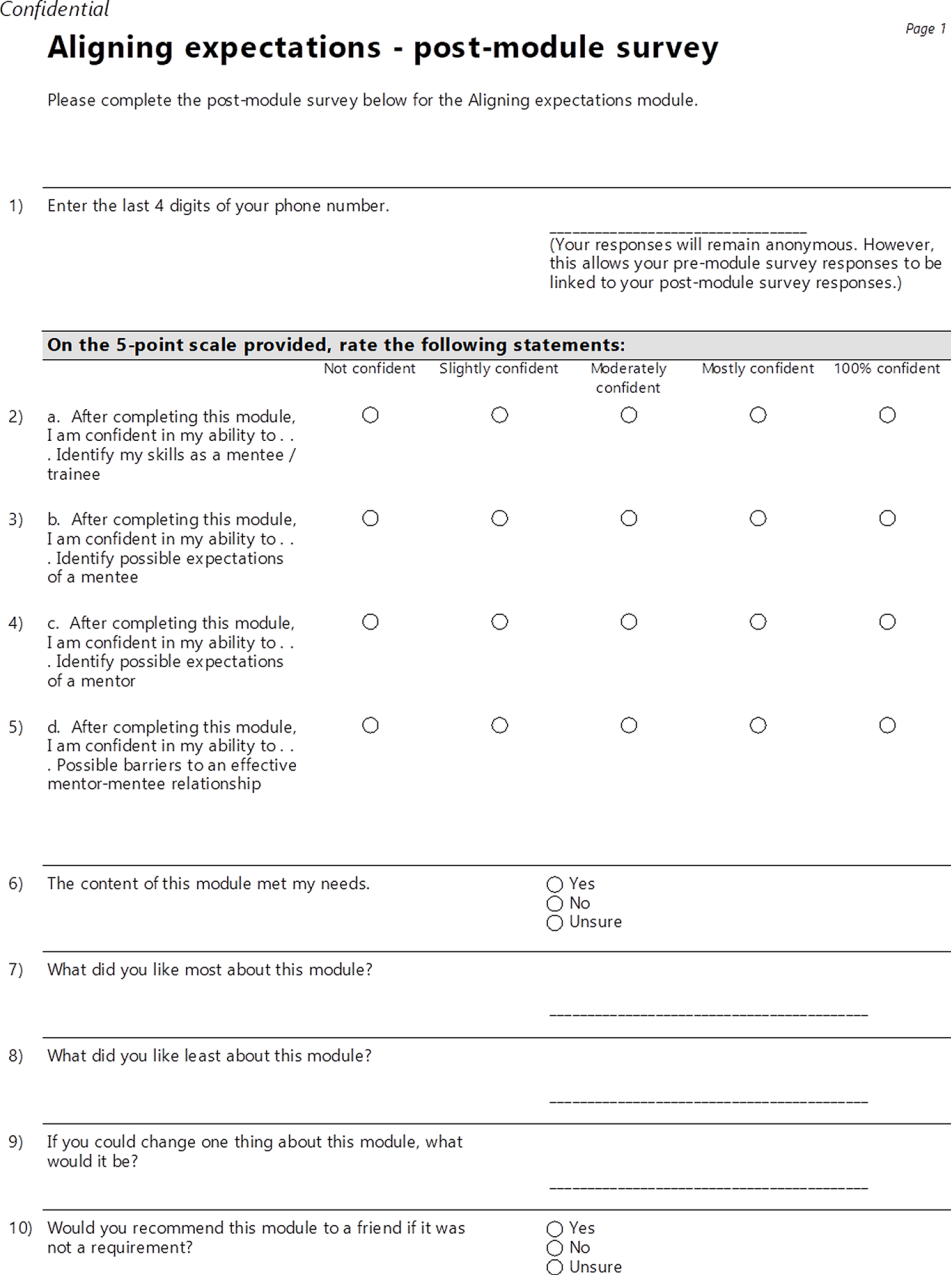
Post-module survey for the *Aligning expectations* module.

To complete a module, participants performed three tasks. Task #1 was located at the beginning of each module and was labeled as the pre-module survey. This pre-module survey was completed before reviewing any of the assignments within a module. Task #2 was to complete all assignments within a module. At the conclusion of each module – after completing all required assignments – Task #3 was to complete the post-module survey. With the exception of *Getting started with Research 101*, each module included a pre-module survey and a post-module survey as required elements. As shown in Fig. 2, the pre-module survey included questions based on the learning objectives with responses provided on a five-point Likert scale. For instance, “I am confident in my ability to … identify my skills as a mentee/trainee” (which corresponds to learning objective #1 for the *Aligning Expectations* module) or “I am confident in my ability to … describe possible barriers to an effective mentor-mentee relationship” (learning objective #4). As shown in Fig. 3, the post-module survey included the same questions based on the learning objectives as the pre-module survey, as well as additional open-ended text field questions: 1) *The content of this module met my needs*; 2) *What did you like most about this module?*; 3) *What did you like least about this module?;* 4) *If you could change one thing about this module, what would it be?*; and 5) *Would you recommend this module to a friend if it was not a requirement?*.

Survey data were collected and managed using REDCap electronic data capture tools hosted at the University of Cincinnati. REDCap – Research Electronic Data Capture – is a secure, web-based software platform designed to support data capture for research studies, providing: 1) an intuitive interface for validated data capture; 2) audit trails for tracking data manipulation and export procedures; 3) automated export procedures for seamless data downloads to common statistical packages; and 4) procedures for data integration and interoperability with external sources [7]. The qualitative dataset was analyzed using an inductive content analysis approach that offers a systematic and objective method for classifying words and phrases into meaningful categories, a process which then allows the analysts to distill key ideas from a larger body of text [8].

Paired t-tests were conducted in SAS 9.4 to analyze quantitative changes in Likert scale responses by testing whether the mean change from the pre- and post-module surveys were significantly different than zero.

Once an operational draft of the *Research 101* series was available in Canvas, the overall format and module contents were discussed during multiple online meetings with the CCTST Translational Workforce Development team, as well as leadership of the Medical Student Summer Research Program (MSSRP) and the Summer Medical Student Respiratory Research Fellowship (SMURRF) program. Additionally, interested faculty and staff were registered for *Research 101* so that they could review the modules and provide input.

*Research 101* was piloted during the summer of 2021 with first-year medical students participating in two NIH T35-funded medical student training programs – MSSRP and SMURRF – at the UCCOM. MSSRP and SMURRF students were required to complete all modules with the exception the *Submitting an Institutional Review Board (IRB) protocol at UC* module which was not required for non-UCCOM students. A final quiz was required that consisted of 25 multiple choice questions covering the materials from all required modules. There was no time limit for the quiz, and participants had access to all *Research 101* content during the quiz. Feedback on correct responses was provided. The final quiz was not used as a grade; rather, the final quiz score was utilized for reporting purposes only and to assist in refinement of module content.

The University of Cincinnati Institutional Review Board reviewed the study and determined the research qualified as minimal risk to participants and was exempt from most of the requirements of the Federal Policy for the Protection of Human Subjects.

## Results

During a 6-week period in the summer of 2021, 27 first-year medical students – including 15 in the MSSRP and 12 in the SMURRF programs – were required to complete all elements of *Research 101*. Twenty students were from the UCCOM, while seven were non-UCCOM students.

Pre-module survey results are shown in Table 2. As assessed by mean survey responses, prior to completing the modules, learners were most confident with the *Conducting a literature search* module (3.88–4.04) and least confident with the *Submitting an Institutional Review Board (IRB) protocol at UC* module (1.88–2.38). Mean responses ranged from 2.94 to 4.08 for all other modules and questions. Overall, post-module mean scores were significantly increased compared to pre-module scores (*P* < 0.05) for all modules and questions. The response to “The content of this module met my needs” was high across all modules with 236 (84.0%) “yes” responses, 12 (4.3%) “no” responses, and 33 (11.7%) “unsure.” “No”/“unsure” responses were highest for the *Submitting an Institutional Review Board (IRB) protocol at UC* (7 of 22) and *Study design and data analysis basics* (6 of 22) modules. The mean response to “Would you recommend this module to a friend if it was not a requirement?” was lower with 138 (49.8%) “yes” responses, 57 (20.6%) “no” responses, and 82 (29.6%) “unsure.” “No”/“unsure” responses were highest for the *Introduction to research* (20 of 26), the *Identifying a research mentor and a research project* (17 of 26), and the *Submitting an Institutional Review Board (IRB) protocol at UC* (17 of 21) modules.

**Table 2.** Pre-module and post-module survey results for the *Research 101* modules

Module/question	Pre-module mean	Post-module mean
**Introduction to research/**I am confident in my ability to …		
1. Define research	3.61	4.36
2. Identify various types of research	3.68	4.29
3. Recognize the stages of clinical/translational research	2.94	4.14
4. Identify the steps of the scientific method	3.39	4.29
The content of this module met my needs (yes/no/unsure)	–	24/0/2
Would you recommend this module to a friend if it was not a requirement? (yes/no/unsure)	–	6/10/10
**Aligning expectations/**I am confident in my ability to …		
1. Identify my skills as a mentee/trainee	3.57	4.14
2. Identify possible expectations of a mentee	3.57	4.29
3. Identify possible expectations of a mentor	3.46	4.14
4. Describe possible barriers to an effective mentor–mentee relationship	3.46	4.22
The content of this module met my needs (yes/no/unsure)	–	21/2/3
Would you recommend this module to a friend if it was not a requirement? (yes/no/unsure)	–	11/8/7
**Identifying a research mentor and a research project/**I am confident in my ability to …		
1. Describe the characteristics of your ideal mentor	3.75	4.25
2. List resources to identify an appropriate mentor and to develop a research project	3.32	4.29
3. Strategize possible solutions to common barriers to an effective mentor–mentee relationship	3.36	4.07
4. Understand how to write a clear and concise research project description	3.25	4.18
The content of this module met my needs (yes/no/unsure)	–	21/0/5
Would you recommend this module to a friend if it was not a requirement? (yes/no/unsure)	–	9/9/8
**Introduction to human subjects research and protections/**I am confident in my ability to …		
1. Understand the history of human subjects research	3.29	4.32
2. Define human subjects research	3.61	4.36
3. List the requirements for protecting human subjects in research	3.21	4.36
The content of this module met my needs (yes/no/unsure)	–	24/0/2
Would you recommend this module to a friend if it was not a requirement? (yes/no/unsure)	–	19/3/4
**Submitting an Institutional Review Board (IRB) protocol at UC/**I am confident in my ability to …		
1. List resources for submitting an IRB protocol for review	1.88	3.50
2. List the components of an IRB submission	2.04	3.38
3. Understand common issues with IRB submissions and strategize possible solutions	2.08	3.22
4. List resources for the protection of human subjects, biosafety, and animal care and training on these topics	2.38	3.42
The content of this module met my needs (yes/no/unsure)	–	15/3/4
Would you recommend this module to a friend if it was not a requirement? (yes/no/unsure)	–	4/6/11
**Conducting a literature search/**I am confident in my ability to …		
1. Describe the use of PubMed for literature searches	4.00	4.46
2. Describe the use of Google Scholar for literature searches	4.04	4.46
3. List resources for conducting an effective literature search	3.88	4.42
The content of this module met my needs (yes/no/unsure)	–	21/1/4
Would you recommend this module to a friend if it was not a requirement? (yes/no/unsure)	–	17/2/6
**Effective writing for publication/**I am confident in my ability to …		
1. List the various types of publications	3.62	4.31
2. Describe the elements of a manuscript	3.42	4.31
3. Describe an effective abstract	3.58	4.27
4. List resources for improving ones writing skills	3.38	4.19
The content of this module met my needs (yes/no/unsure)	–	24/2/0
Would you recommend this module to a friend if it was not a requirement? (yes/no/unsure)	–	14/3/9
**Transparency, rigor, and reproducibility in research/**I am confident in my ability to …		
1. Describe the need for transparency and its impact on the research process	4.04	4.46
2. Describe the need for rigor and reproducibility and their impact on the research process	4.08	4.52
3. Identify approaches to enhance the transparency, rigor, and reproducibility of your research project	3.65	4.48
The content of this module met my needs (yes/no/unsure)	–	22/0/3
Would you recommend this module to a friend if it was not a requirement? (yes/no/unsure)	–	13/4/8
**Study design and data analysis basics/**I am confident in my ability to …		
1. List different types of epidemiologic studies	3.04	4.31
2. Define key characteristics and limitations of cross-sectional studies	3.50	4.31
3. Define key characteristics and limitations of cohort studies	3.46	4.27
4. List resources for study design and data analysis	3.31	4.19
The content of this module met my needs (yes/no/unsure)	–	20/2/4
Would you recommend this module to a friend if it was not a requirement? (yes/no/unsure)	–	15/6/4
**Presenting your summer research/**I am confident in my ability to …		
1. Describe elements of an effective poster	3.54	4.35
2. List venues for presenting research projects	3.19	4.23
3. Describe elements of an effective lab meeting presentation	3.42	4.15
The content of this module met my needs (yes/no/unsure)	–	23/1/2
Would you recommend this module to a friend if it was not a requirement? (yes/no/unsure)	–	14/3/8
**Evaluating the literature and presenting a journal club/**I am confident in my ability to …		
1. Describe strategies for reading and critiquing a scientific article	3.69	4.27
2. Describe elements of an effective journal club	3.31	4.12
The content of this module met my needs (yes/no/unsure)	–	21/1/4
Would you recommend this module to a friend if it was not a requirement? (yes/no/unsure)	–	12/4/9

Content analysis of the qualitative data generated by open-ended text responses from the post-module surveys revealed overall themes in student responses to the *Research 101* modules. For the *What did you like most about this module* question, these included 1) shorter videos that present information in an easily understood and visual manner, 2) ability to self-pace, 3) case studies, and 4) practical, relevant examples (e.g., IRB, UC Radiation study, and research misconduct) and activities (e.g., finding an article). For the *What did you like least about this module* and the *If you could change one thing about this module, what would it be* questions, themes included 1) technical issues (e.g., audio problems, links that did not work/videos would not load), 2) general course organization (e.g., clear directions to accompany website links; better flow of module materials; too much movement between course activities, and changes to the discussion board requirement), 3) timing of the modules (e.g., many felt they could have used this information before starting their summer programs, some of the modules felt redundant), and 4) course materials (e.g., more specific examples, case studies, activities rather than passive knowledge transmission, shorter videos, bulleted information sheets to summarize major points of each module).

The median final quiz score was 21 out of a possible 25 and ranged from 16 to 24.

## Discussion

Research training is important for learners at many different levels, and resources to provide this training in a comprehensive, student-centered manner are highly desirable. The pilot data demonstrate significant learning resulting from completion of *Research 101*, as post-module mean scores were significantly higher than pre-module results for all modules and questions. Final quiz scores were good but also highlighted opportunity for additional student learning.

We estimate that this pilot of *Research 101* required ∼10–12 h to complete. While students did not express any concerns in the surveys about this time commitment, the current scope of *Research 101* has been limited to avoid additional student time for completion, since this has been structured as a zero-credit option outside of the regular medical school curriculum. For the instructor, once the content and framework of *Research 101* have been established, additional time is needed each year to 1) revise module(s) content based on participant feedback and the availability of online educational materials or the creation of new materials, 2) fix technical issues with external hyperlinks and/or survey administration, 3) monitor participant progress in real time if needed, and 4) discuss *Research 101* content and access to program directors that may be interested in utilizing it in the future.

Qualitative and quantitative responses provide several additional opportunities for future iterations of *Research 101*. For summer 2022, *Research 101* has been expanded to include another medical student scholarly research program that will engage additional students. Other changes made to *Research 101* include streamlining the assignments for each module, queueing the pre- and post-module surveys to limit the number of clicks required to complete each module, creation of a Table of Contents to enhance user accessibility, and addition of a new module entitled *Race and racism in research and medicine*.

The academic medical community has been concerned about the reluctance of young physicians to prepare for and undertake careers in research for several decades now [9–11]. Houlden *et al*. found that after completion of a mandatory research elective in the second year of medical school, there was a significant increase in students interested in pursuing research careers [12]. However, previous research has identified significant barriers to students pursuing research training during medical school, such as lack of institutional incentives to conduct research, lack of infrastructure, insufficient access to faculty mentors, lack of awareness of research opportunities occurring locally, and the absence of a research office or coordinator of training [13,14]. In a survey of US medical students, only 19.4% reported having a mandatory course on research methods, while 28.7% reported that an elective research course was available at their institution [14]. *Research 101* offers an important solution to some of the known barriers to effective medical student research education in that it provides a structured introduction to key research topics in a highly accessible format. Additionally, it offers a basic training infrastructure that unifies student research training in a coherent and sequential framework that is flexible enough to be adapted to unique program or student needs.

This study has several limitations to consider. First, *Research 101* was piloted initially with a relatively small number of medical students. However, we intend to continuously evaluate *Research 101* over time as it is expanded to enroll larger numbers of programs and students, including research staff and clinical research professionals as well. Second, there may be selection bias among the students who completed this pilot. Since the pilot focused on students who had already chosen to pursue summer research experiences, these students might be more naturally inclined to have research interests or had previous research experience prior to starting *Research 101*. Despite this potential bias, the breadth of evaluation data collected proved helpful to improving the methods and materials included in *Research 101* for future audiences that may not have a predisposition to or background in research. Third, learners had to complete the preceding module before moving to the next module. However, optional modules are easily accommodated by Canvas, as are modules that certain groups of learners – but not all learners – must complete. For instance, medical students may be required to complete modules that are not required for graduate students, or one section of *Research 101* may include additional modules that are program-specific and not required for students enrolled in other sections of *Research 101*. Fourth, due to the extensive use of existing resources (e.g., websites, case studies, and videos), these links and resources must be re-evaluated each time *Research 101* is offered. Finally, some topics may require more direct interaction with students. Research 101 is not designed to replace direct, in-person interactions, but rather to provide an additional option for students to learn given distinct learning styles, limited space for new content within the existing medical school curriculum, and the varying interests of students.

If we, as medical educators, wish to increase and improve the training that medical students receive on biomedical research methods and processes, understanding the obstacles that prevent them from pursuing such training is a critical first step. Training that is asynchronous and widely available, but also organized within a learning management system where students can engage with the material on their own time and complete course milestones in a logical, cumulative order can help overcome some of the obstacles students have reported in the literature. *Research 101* is a comprehensive overview of important topics that are intended to compliment the learning environment for students who are preparing for or already conducting research projects and not a substitute for a hands-on research experience. Based on this pilot experience, we believe the *Research 101* series is a valuable addition to the summer medical student research experience which can also be adopted and adapted by other medical student programs in other institutions.
